# Effect of composite conjugated materials on tissue healing during exercise rehabilitation training

**DOI:** 10.3389/fchem.2023.1279463

**Published:** 2023-10-19

**Authors:** Jie Li, Jing Zhang

**Affiliations:** ^1^ College of Physical Education, Gannan Normal University, Ganzhou, Jiangxi, China; ^2^ Orthopedics, Hubei Provincial Hospital of TCM (Affiliated Hospital of Hubei University of Chinese Medicine), Wuhan, Hubei, China

**Keywords:** composite conjugated materials, sports rehabilitation training, tissue healing, skin healing, inflammatory response

## Abstract

The application of traditional materials to tissue healing in sports rehabilitation training has problems such as poor effect, high rejection reaction, and slow healing speed. It also brings many challenges to the development of sports rehabilitation training. This article aims to explore the impact of composite conjugated materials on tissue healing to promote rapid and efficient tissue healing and improve the effect of sports rehabilitation training. Through research and analysis, this article found that composite conjugated materials have unique biocompatibility and can promote cell growth and differentiation. In skin tissue healing, composite conjugated materials can control the release rate and duration of drugs to promote skin healing. During the fracture healing process, conjugated materials can provide growth factors and extracellular matrix components, stimulate bone cell proliferation and differentiation, and promote fracture healing. In terms of soft tissue injuries, composite conjugated materials serve as supporting structures or matrices, providing a favorable environment for the regeneration of damaged tissue. In the regulation of inflammatory responses, composite conjugated materials reduce inflammatory responses and accelerate the healing process by modulating immune responses. The results of this study show that 1 week after the experiment, the skin healing rates of the control group and the experimental group were 42.55% and 58.17% respectively; 5 weeks after the experiment, the skin healing rates of the control group and the experimental group were 51.28% and 73.24% respectively. After 1, 2, 3, 4, and 5 weeks of experiment, it was found that the average tissue repair rates of the control group were 44.03%, 54.18%, 58.40%, 67.08%, and 72.09% respectively, and the average tissue repair rates of the experimental group were 52.18%, 61.91%, 63.40%, 74.61%, and 85.05% respectively. This study highlights the huge potential of composite conjugated materials in promoting tissue healing and tissue repair, and is of great significance for promoting technological progress in the field of sports rehabilitation and improving rehabilitation effects.

## 1 Introduction

Tissue healing is a crucial step in restoring normal function of damaged tissues during exercise rehabilitation training. Through tissue healing, damaged muscles, bones, joints, or other tissues can gradually be repaired and rebuilt, thereby restoring the function of the damaged area. If the tissue heals slowly, the relevant muscles, bones, or joints may not be able to effectively restore their normal function, resulting in functional loss; in severe cases, it may lead to disability. For sports rehabilitation training, promoting tissue healing requires the interaction between materials and damaged tissues, and traditional materials often cannot provide this biological activity, thereby limiting the speed and effectiveness of tissue healing. Composite conjugated materials have good biocompatibility and biological activity. This material can provide mechanical support and promote cell adhesion and proliferation; it can release bioactive molecules and promote tissue growth and repair, which is expected to become a new strategy for promoting tissue healing in sports rehabilitation training.

Exercise rehabilitation training is of great significance for restoring function, reducing pain, preventing complications, promoting fiber reconstruction, and improving exercise performance. In order to explore the intervention value of implementing somatosensory interactive rehabilitation training on patients with humeral fractures in their rehabilitation process, Li Jiankang selected 60 patients with humeral fractures admitted from December 2018 to December 2020; the control group received routine rehabilitation training, while the observation group received somatosensory interactive rehabilitation training. It was found that the implementation of somatosensory interactive rehabilitation training for humerus fracture patients can effectively shorten the fracture healing time, and can improve the function of shoulder joint mobility while relieving pain (Li and Zhao, 2021). In order to observe the application effect of early rehabilitation training in the rehabilitation care of limb fractures, Guo Jianping selected 90 cases of limb fractures who received treatment from August 2018 to May 2019. The first 45 cases were treated as the control group for routine care, and the last 45 cases were treated as the observation group for early rehabilitation training. The conclusion indicated that the implementation of early rehabilitation training has positive significance in promoting the healing of limb fractures, preventing various complications, improving motor function and quality of life (Guo, 2020). In order to explore the application effect of nursing intervention combined with somatosensory interactive rehabilitation training based on the integrated theory of health behavior change in traumatic lower limb fractures, Xiao Hongli selected 80 patients with traumatic lower limb fractures from January 2019 to February 2021. The control group received routine nursing combined with somatosensory interactive rehabilitation training, while the observation group received nursing interventions based on the integrated theory of health behavior change on the basis of the control group. The results indicated that nursing interventions based on the integrated theory of health behavior change combined with somatosensory interactive rehabilitation training can promote the recovery of lower limb function (Xiao, 2023). Sports rehabilitation training can accelerate the rehabilitation process and promote tissue healing and functional recovery, but there is currently limited research on the impact of composite conjugated materials on tissue healing during sports rehabilitation training.

Composite conjugated materials provide a promising therapeutic option for sports rehabilitation training, helping to accelerate the rehabilitation process and improve rehabilitation outcomes. In order to achieve bone defect healing based on biomaterials, Winkler T proposed transferring the lessons learned from bone healing to challenging defect scenarios, and reviewed the endogenous cascades of bone material formation, as well as how these cascades can be transferred to new perspectives in biomaterial driven methods of bone regeneration (Winkler et al., 2018). In order to process biomaterials into a form suitable for bone tissue engineering, Koons Gerry L proposed the design of bone tissue engineering materials. He analyzed the composition and structure of natural bone tissue and appropriately selected biomimetic natural materials, such as polymers, bioceramics, metals, and composite materials, to process them into suitable bone tissue engineering. The results showed that the material has good biocompatibility and can fuse with cells around bone tissue (Koons Gerry et al., 2020). Conventional therapy is difficult to cure sports injuries. In order to provide rehabilitation effects for sports injuries, Looi Qi Hao proposed a new method of using mesenchymal stem cells to treat sports injuries. He found that mesenchymal stem cells can regulate host immune responses and promote angiogenesis, thereby increasing cell migration rates. This method is becoming increasingly popular (Qi et al., 2020). After the above research, it has been found that composite conjugated materials have been widely used in the field of tissue healing, which can promote the rehabilitation process of sports injuries and bring better results for sports rehabilitation training.

Traditional rehabilitation methods mainly include physical therapy and exercise training. Composite conjugated materials, as a new type of auxiliary material, can provide additional means and strategies for sports rehabilitation training. This helps to broaden the scope of rehabilitation methods and provide more options and possibilities to meet the needs of different patients. The goal of exercise rehabilitation training is to restore the patient’s motor function. The use of composite conjugated materials as auxiliary materials can improve the rehabilitation effect. The biological activity of the materials can increase cell adhesion and proliferation, and promote tissue regeneration, thereby accelerating the patient’s rehabilitation process. The composite conjugated materials analyzed in this article contribute to tissue repair and regeneration at the wound site by providing mechanical support and biological activity. By regulating tissue growth and differentiation, composite conjugated materials can promote the healing process of damaged tissues. In the future, this material would receive more applications and development, making greater contributions to the cause of human health.

## 2 Exercise rehabilitation training and tissue healing

### 2.1 Tissue healing process and influencing factors

Tissue healing is a complex physiological process that includes multiple stages such as inflammation, coagulation and vascular reconstruction, cell proliferation, and repair stages. In the normal healing process, each stage alternates to achieve the goal of tissue recovery. However, if there is a problem in a certain stage during the healing process, it would affect the overall healing of the organization.

Inflammation stage: in the first few days after tissue injury, the inflammatory response is initiated and blood vessels dilate, causing blood and cytokines to flow into the injured area. The inflammatory response helps eliminate pathogens and waste, but excessive or chronic inflammation may have a negative impact on healing.

Blood coagulation and vascular reconstruction stage: coagulation factors help tissues form blood clots on the wound, after which new blood vessels begin to grow (angiogenesis) to supply nutrients and oxygen, and take away metabolic products.

Cell proliferation and repair stage: in the wound, various types of cells begin to proliferate and migrate to fill the defect area, thus forming new tissue. Fibroblasts produce collagen to establish structural support. Epidermal cells begin to divide and gradually restore the integrity of the epidermal layer.

Reshaping stage: in this stage, newly formed organizations would gradually reshape and repair. Collagen is rearranged to enhance the strength and durability of the tissue. Due to the reconstruction of cells and matrix, the wound gradually contracts and ultimately forms scar tissue.

The factors that affect tissue healing include wound severity, infection, ischemia or poor blood supply, lack of nutrition, abnormal scar formation, etc. If problems occur during the tissue healing process, it may affect the entire healing process, prolong the recovery time, or lead to abnormal healing. Larger or deeper wounds may take longer to recover and may lead to more obvious scar formation. If the wound is infected, the inflammatory response may intensify and the healing process may be disrupted.

In sports rehabilitation training, professional medical personnel usually pay attention to these issues and take appropriate measures to promote normal tissue healing. This may include regular replacement of dressings, provision of appropriate nutritional support, physical therapy and rehabilitation training, and monitoring of wound infections and scars.

### 2.2 Composite conjugated materials

Composite conjugated materials refer to composite materials composed of two or more materials, at least one of which is an organic semiconductor material. This material has good photoelectric properties and chemical stability, and is suitable for use in the biomedical field (Andia et al., 2019; Christman, 2019). In recent years, composite conjugated materials have also been widely used in tissue engineering, cell culture, drug delivery, and other fields (Cao et al., 2019; Lv et al., 2022). At present, researchers have applied composite conjugated materials to research on tissue healing. By attaching composite conjugated materials to wounds, they can effectively promote tissue healing and reduce the risk of infection and scars. In addition, composite conjugated materials can promote angiogenesis and accelerate wound healing.

Composite conjugated polymer materials are typically composed of conjugated polymers and other biocompatible materials such as proteins, peptides, or other polymers. The conjugated polymers used in these materials are typically composed of repeating units containing conjugated double bonds, which give them unique electronic and optical properties.

(1) Organic-inorganic composite conjugated materials

**FIGURE 1:**
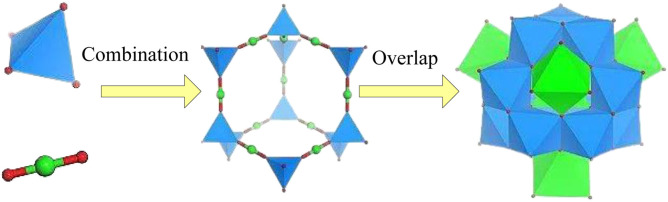
Schematic diagram of molecular combination overlap.

Organic-inorganic composite conjugated materials are composite materials composed of organic conjugated molecules and inorganic nanomaterials. Organic molecules can provide conjugation properties, while inorganic nanomaterials can form conductive, thermal, and optoelectronic properties in composite materials. Due to the different selection and regulation of organic and inorganic components, the performance and characteristics of composite materials can be adjusted by changing component ratios, interface interactions, and adding functionalized molecules to achieve the adjustment of material properties and meet different application needs (Cao et al., 2022; Deng et al., 2022). Taking organic/metal oxides as an example, organic molecules are conjugated aromatic compounds, such as polyaniline, while metal oxides are typically nanomaterials such as zinc oxide and copper oxide. In composite materials, π electron sharing and overlap occur between organic molecules and metal oxides, which enables rapid electron transfer in the material, significantly improving the conductivity and optoelectronic properties of the material. The schematic diagram of molecular combination overlap is shown in Figure 1.

The addition of inorganic components can improve the thermal stability of organic materials. Organic-inorganic composite conjugated materials can maintain stability under high temperature conditions and resist photodegradation and thermal degradation, which gives them advantages in high-temperature applications. The addition of inorganic nanoparticles or nanomaterials can enhance the mechanical strength and rigidity of organic materials, thereby interfering with the improvement of the material’s tensile and impact resistance. This makes organic-inorganic composite conjugated materials have promising applications in flexible electronics, sensors, and structural materials.

(2) Hybrid composite conjugated materials

Hybrid composite conjugated materials are usually formed through chemical bonding of conjugated structural units, thus they can form conductive, thermal, optoelectronic and other characteristics in composite materials (Zeng et al., 2018; Chen et al., 2020). Hybrid conjugated composite materials have excellent mechanical properties and biocompatibility, and can serve as carriers for supporting and maintaining damaged tissue structures. For example, in fracture rehabilitation, applying hybrid composite conjugated materials to the fracture site can provide stable support and promote fracture healing. By providing support for cell adhesion and growth to promote the formation of new tissues, these materials typically have high porosity and good permeability, providing an ideal growth environment for cells and accelerating tissue repair and regeneration at the site of trauma.

Currently, common hybrid conjugated materials include π conjugated small molecules and polymers, as well as organic/inorganic hybrid conjugated materials. Among them, π conjugated small molecules and polymers are bonded to form composite materials, which can significantly improve the conductivity and photoelectric properties of the material. Organic/inorganic hybrid conjugated materials are composite materials composed of organic conjugated molecules and inorganic conjugated molecules, which are formed by chemical bonding between them.

(3) Bio-inorganic composite conjugated materials

Bio-inorganic composite conjugated materials are composite materials composed of biomolecules and inorganic nanomaterials, which typically provide biocompatibility through biomolecules.

**FIGURE 2:**
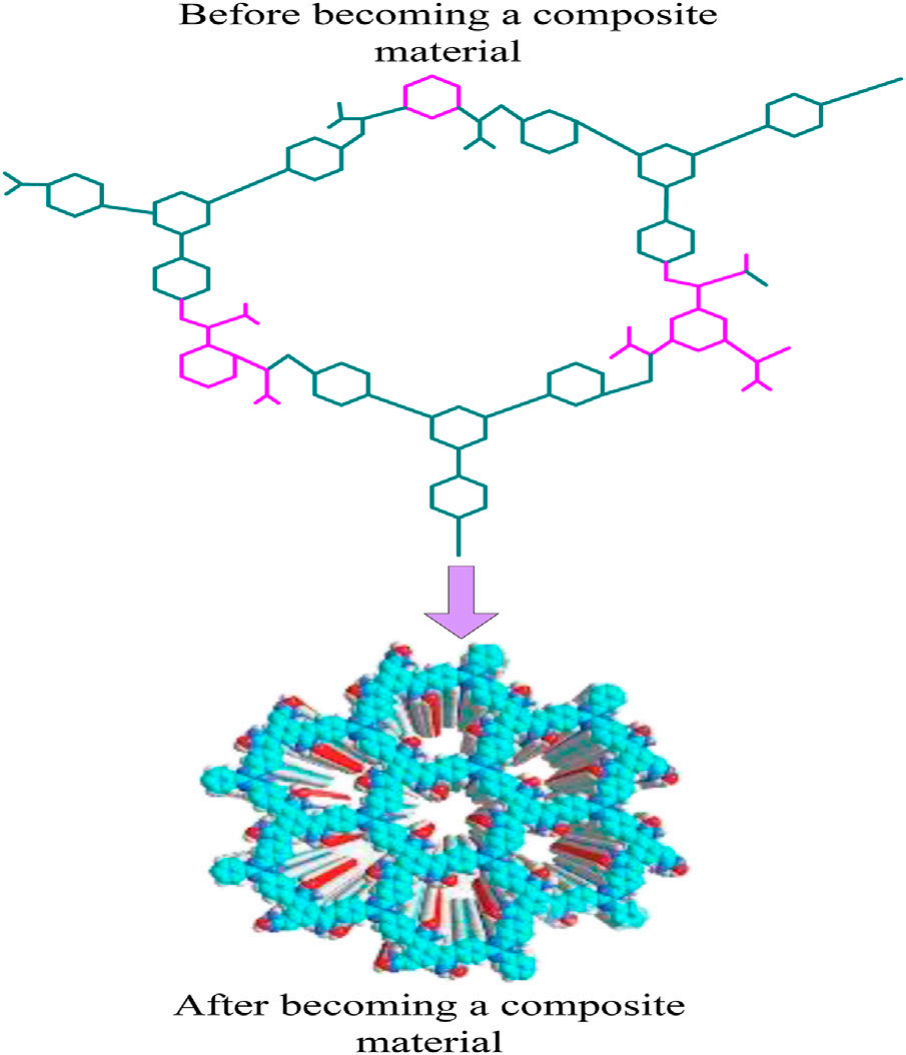
Schematic diagram of composite materials.

Bio-inorganic composite conjugated materials include protein/metal nanomaterials, biomolecules/metal oxides, etc. Taking protein/metal nanomaterials as an example, protein is an organic molecule with biocompatibility, while metal nanomaterials are inorganic materials with conductivity, thermal conductivity, and other characteristics. The interaction between protein molecules and metal nanomaterials in composite materials results in good biocompatibility and conductivity. At the same time, this composite material can also be applied in biosensors, biological imaging, and other fields. The schematic diagram of composite materials is shown in Figure 2.

In Figure 2, before becoming a composite material, the structure was still simple, but after becoming a composite material, the structure became more complex.

Composite conjugated polymer materials have the effect of promoting cell growth and differentiation both *in vitro* and *in vivo*. The unique electronic and optical properties of conjugated polymers can provide clues for cellular behavior, including cell adhesion, proliferation, and differentiation. Conjugated polymers can regulate cellular processes such as gene expression, cellular signaling, and extracellular matrix production. A composite material composed of polythiophene and polycaprolactone (PCL) has been proven to promote the differentiation of mesenchymal stem cells into neural cells (Shang et al., 2021). PCL is a biocompatible and biodegradable polymer widely used in tissue engineering. Composite materials support cell attachment and proliferation, and promote the expression of neural differentiation markers.

Bio-inorganic composite conjugated materials usually use biodegradable materials as carriers, which have good biocompatibility with human tissues and do not cause significant immune reactions or rejection phenomena. Bio-inorganic composite conjugated materials have certain mechanical strength and stability, which can provide the structural support required for damaged tissues. In sports rehabilitation training, these materials can be used to repair fractures, soft tissue injuries, etc. By providing stable support, they can promote tissue healing and repair processes (Wang et al., 2018). The mechanical strength of bio-inorganic composite conjugated materials is:

$$R=\frac{\rho }{S}$$ (1)

The maximum force ρ refers to the maximum tensile force that a material can withstand, while the cross-sectional area S of a material refers to the cross-sectional area in the direction of force. Mechanical strength is commonly used to describe the failure performance of a material under force.

Bio-inorganic composite conjugated materials can promote the adhesion and growth of vascular endothelial cells, and can release angiogenic factors such as vascular endothelial growth factor to stimulate angiogenesis. This helps to improve local blood supply and provide oxygen and nutrients, thereby promoting the repair and regeneration of damaged tissues.

**FIGURE 3:**
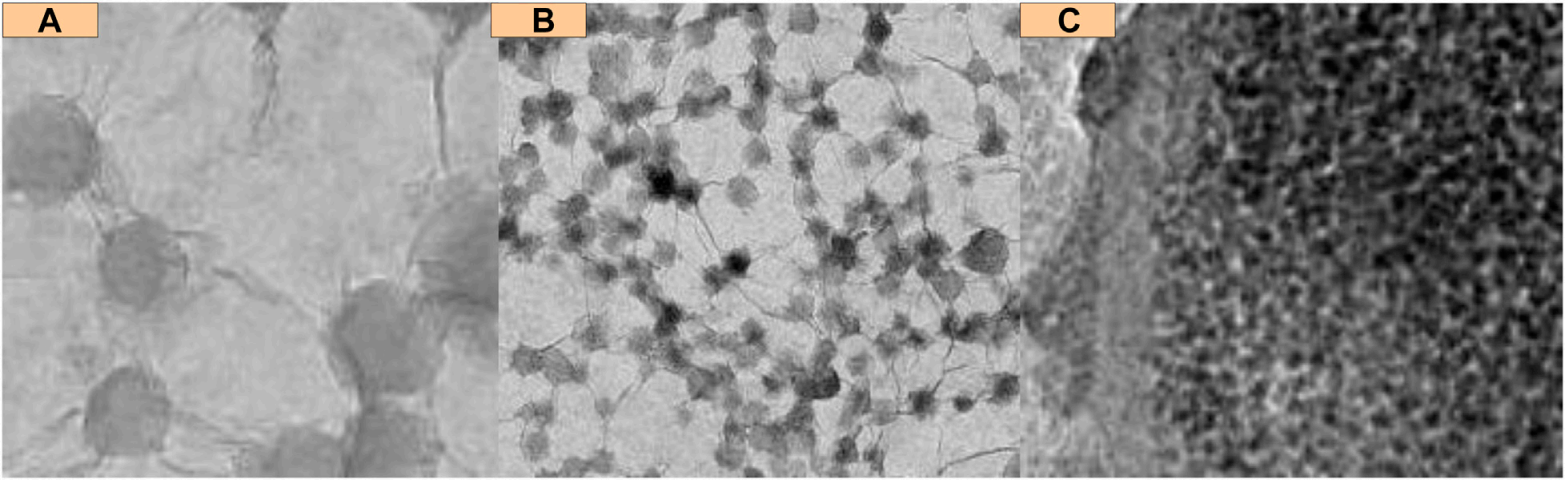
Biocompatibility schematic diagram. **(A)** Original image of cells placed in material **(B)** Image of cells flowing **(C)** Image when cells show fine compatibility.

Composite materials composed of polyaniline and collagen have been proven to promote the proliferation and differentiation of osteoblasts. Polyaniline is a conductive conjugated polymer that has been proven to promote osteogenesis and bone regeneration. Collagen is the main component of bone tissue and is commonly used in tissue engineering applications due to its biocompatibility and ability to support cell growth and differentiation. Composite materials provide a conductive scaffold for cell attachment and proliferation, and promote the expression of osteogenic markers such as alkaline phosphatase and osteocalcin. The schematic diagram of biocompatibility is shown in Figure 3.

In Figure 3A is the original image of cells placed in the material, and b is the image of cells flowing; c is a graph of fine compatibility. It can be seen that the cell giants in the material have biocompatibility.

Biocompatibility is a key consideration for the application of composite conjugated polymer materials in tissue engineering and regenerative medicine. Although conjugated polymers have been proven to have unique properties that promote tissue healing and regeneration, their potential cytotoxicity and immunogenicity must be carefully evaluated.

The swelling rate is used to measure the material’s ability to absorb water. A higher swelling rate means that the material can absorb more water. For tissue engineering and regenerative medicine, a moderate swelling rate helps provide a humid environment and promotes cell growth and tissue repair:

$$S_{w}=\frac{W_{s}-W_{d}}{W_{d}}$$ (2)

Among them, $W_{s}$ is the weight of the water containing composite conjugated material, and $W_{d}$ is the weight of the material in the dry state.

Cytotoxicity refers to the evaluation of the toxicity of a material to cells, which can be calculated by comparing the number of cells in contact with the material with the number of cells not in contact with the material. A higher cell survival rate means that the material has lower toxicity to cells. For tissue engineering and regenerative medicine, the material should have good cell compatibility and would not have harmful effects on surrounding tissues and organs. The formula for evaluating cytotoxicity is:

$$Cell_{i}=\frac{N_{c}}{N_{t}}$$ (3)

Among them, $N_{c}$ represents the number of cells in contact with the material, and $N_{t}$ represents the number of cells not in contact with the material.

In the field of modern medicine, more and more research is focusing on the role of composite conjugated materials in tissue healing. Composite conjugated materials have good biocompatibility and can be used in various tissue engineering and biomedical applications, such as bone tissue engineering, cartilage tissue engineering, cardiovascular medicine, and skin repair.

## 3 Application of composite conjugated materials in sports rehabilitation training

### 3.1 Role of composite conjugated materials in tissue healing

(1) Skin trauma

Skin trauma is one of the most common types of sports injuries in daily life. In the medical field, skin injuries can be divided into shallow trauma and deep trauma. Shallow trauma usually refers to superficial skin damage such as burns, cuts, and abrasions, while deep trauma refers to damage to skin and muscle tissue, such as cuts, collisions, and gunshot wounds. In skin tissue healing, composite conjugated materials can play their role in various ways, and can serve as dressings to promote skin wound healing (Xu et al., 2021; Ijaola et al., 2022). Composite conjugated materials can cover the wound surface and provide a protective barrier to block the invasion of external bacteria, viruses, pollutants, etc., thereby reducing the risk of infection and inflammation. Composite conjugated materials have good permeability and wettability, which can absorb and maintain moisture on the wound surface, forming a humid environment. A humid environment helps promote cell movement, proliferation, and wound healing, while reducing dryness and scabbing of the wound and promoting faster healing.

As a drug carrier, composite conjugated materials can promote wound healing by controlling drug release; by controlling the structure and composition of materials to regulate the release rate and duration of drugs, targeted and sustained therapeutic effects can be achieved (Zahid et al., 2022). The drug release rate is calculated by measuring the mass and surface area of the drug released within a given time. The drug release rate is:

$$D_{r}=\frac{M}{A}\times t$$ (4)

Among them, M is the mass of the dissociated drug, and A is the surface area of drug release; t is the time. The calculation of material release rate helps to control the release of drugs on the wound surface, thereby achieving sustained and targeted therapeutic effects. The promoting effect of composite conjugated materials on the migration of skin wound cells can be expressed as cell migration rate $Cell_{m}$ :

$$Cell_{m}=\frac{D_{j}}{t}$$ (5)

Among them, $D_{j}$ is the distance of cell migration, and t is the time. The higher cell migration rate means that composite conjugated materials can effectively promote cell movement and wound closure.

Composite conjugated materials can regulate cell activity and promote cell proliferation and differentiation, thereby accelerating wound closure and regeneration. The components in certain composite conjugated materials can release bioactive substances such as growth factors to stimulate cell activity, thereby promoting wound healing. Composite conjugated materials promote tissue healing by increasing cell proliferation and the formation of new blood vessels, and prevent wound infection and scar formation.

(2) Fracture healing

Fracture healing is the process of bone tissue regeneration after a fracture, in which composite conjugated materials can promote the regeneration and repair of bone tissue. Some components in composite conjugated materials can promote the proliferation and differentiation of bone cells, while also preventing infection and osteolysis. Some components in composite conjugated materials have antibacterial and antimicrobial properties, which can prevent infection and maintain a clean environment for wounds.

Some components in composite conjugated materials may contain growth factors, such as bone morphogenetic protein (BMP) and fibroblast growth factor (FGF). These growth factors have specific molecular structures that can bind to receptors on bone cells and activate the process of cell proliferation and differentiation through signaling pathways. For example, BMP can bind to receptors on the cell membrane and initiate signaling pathways within the cell, thereby promoting the proliferation and differentiation of bone cells. The general relationship between bone cell proliferation and differentiation is:

$$Cell_{y}=\left(Growth_{F}+E_{m}\right)*Other_{F}$$ (6)

Among them, $Growth_{F}$ represents growth factor; $E_{m}$ represents the extracellular matrix component; $Other_{F}$ represents other factors that may affect the proliferation and differentiation of bone cells.

Other components in composite conjugated materials may include extracellular matrix (ECM) components, such as collagen and hyaluronic acid. These molecules have specific structures that can provide scaffold structure and physical support, and promote cell attachment, migration, and proliferation. In addition, ECM components can also interact with receptors on the cell surface to regulate cell function and behavior. Some components in composite conjugated materials may have antibacterial activity, such as silver ions, antibiotics, etc. The molecular structure of these components can exist in different forms, such as ionic or compound forms. Silver ions can be released into the surrounding environment and interact with bacterial proteins and deoxyribonucleic acid to disrupt the biological function of bacteria, thereby achieving antibacterial effects.

(3) Soft tissue injury

Soft tissue injury refers to the damage to soft tissues such as ligaments, muscles, tendons, and joints. In the treatment of soft tissue injury, composite conjugated materials also play an important role. Composite conjugated materials can serve as scaffolds and fillers to promote the regeneration and repair of soft tissue, and provide the necessary support and growth environment for soft tissue, thereby promoting the regeneration and repair of soft tissue. Compared with traditional materials, composite conjugated materials have better adhesion and biocompatibility.

Composite conjugated materials provide a favorable environment for the regeneration and repair of damaged tissues. The special structure and composition of the materials can simulate the characteristics of natural tissues and promote cell attachment, migration, and proliferation, thereby accelerating the repair process of damaged tissues. By measuring the area size of damaged tissue after a certain period of time and comparing it with the initial size, the percentage of tissue repair rate can be calculated. The higher tissue repair rate means that composite conjugated materials can accelerate the repair process of damaged tissues. The tissue repair rate can evaluate the effect of composite conjugated materials on the repair rate of damaged tissues:

$$Rate_{a}=\frac{A_{2}-A_{1}}{A_{1}}$$ (7)

$A_{1}$ is the initial size of the damaged tissue area, and $A_{2}$ is the area size of the damaged tissue after a certain period of time.

Composite conjugated materials have extensive application value in tissue healing, as they can promote wound healing in various ways and help patients recover their health as soon as possible. In the future, with the continuous emergence of new materials, the application prospects of composite conjugated materials would be wider.

### 3.2 Application of composite conjugated materials in the regulation of inflammatory response

Inflammatory response is a physiological response of the body to external stimuli, whose main function is to protect the body from harmful substances and eliminate or reduce damage. However, if the inflammatory response is excessively activated or sustained for a long time, it can lead to uncontrolled inflammatory response and produce adverse physiological and pathological effects, leading to various diseases such as inflammatory bowel disease, arthritis, cardiovascular disease, neuritis, etc. The traditional methods of regulating inflammatory response mainly use drugs such as hormones and non-steroidal anti-inflammatory drugs. Although these drugs have achieved certain clinical effects, they have side effects, susceptibility to drug resistance, and may also cause damage to the body during long-term use.

Composite conjugated materials may contain components with medicinal properties, such as nonsteroidal antiinflammatory drugs (NSAIDs), hormonal drugs, etc. The molecular structure of these drugs can reduce inflammation by regulating immune responses. For example, non steroidal anti-inflammatory drugs can inhibit cyclooxygenase, thereby reducing the synthesis of prostaglandins and reducing inflammation and pain. In addition, hormone drugs can inhibit the production and release of inflammatory factors by regulating the expression of inflammation related genes. The components in composite conjugated materials may also contain some bioactive substances, such as peptides, proteins, etc. The molecular structure of these substances can regulate immune responses and inflammatory processes by binding to specific cellular receptors. Some peptides have anti-inflammatory effects and can bind to receptors on the cell surface to activate signaling pathways, thereby reducing inflammation and pain (Fan et al., 2020).

Hybrid conjugated composite materials can serve as carriers for drugs and growth factors, and control their release rate and dosage. In sports rehabilitation training, appropriate drugs or growth factors can be injected into hybrid composite conjugated materials and implanted into damaged areas to promote tissue repair, reduce inflammatory reactions and accelerate the healing process (Zhou et al., 2021; Safina et al., 2022). During the process of exercise rehabilitation, inflammatory reactions are inevitable. Hybrid conjugated composite materials can inhibit the release of inflammatory factors by regulating immune responses, and can alleviate inflammation, pain, and discomfort, thereby providing favorable conditions for tissue healing.

### 3.3 Potential of composite conjugated materials in tissue regeneration and repair

Muscle injury is the most common type of sports injury among athletes and enthusiasts. Currently, composite conjugated materials are a relatively mature therapeutic material. This composite conjugated material not only supports and promotes tissue regeneration, but also avoids subsequent effects on the human body through biodegradation (Gabbett Tim et al., 2021; Zhu et al., 2021). Composite conjugated materials can promote the generation of new blood vessels and improve the blood supply to tissues. Bioactive substances or growth factors can induce the proliferation and differentiation of vascular endothelial cells, and provide signals and support for angiogenesis. This helps to increase the oxygen and nutrient supply to damaged tissues, thereby promoting tissue regeneration and repair. By comparing the number of new blood vessels around the composite conjugated material and in the control group, the angiogenesis index $K_{n}$ is calculated:

$$K_{n}=\frac{l_{c}}{l_{t}}$$ (8)

$l_{c}$ is the number of new blood vessels, and $l_{t}$ is the number of existing blood vessels. A higher angiogenesis index indicates that composite conjugated materials can promote the formation and development of blood vessels.

Composite conjugated materials can be carriers with sustained-release functions, gradually providing therapeutic effects by controlling the release rate and amount of drugs or bioactive substances. The molecular structure of these sustained-release systems can be achieved through polymer membranes, microcapsules, and other forms. For example, polymer membranes in composite conjugated materials can have a slower degradation rate, resulting in slow drug release and sustained promotion of tissue repair.

## 4 Effect of composite conjugated materials on tissue healing

### 4.1 Experimental samples

Mice are the most common experimental animals, with relatively low costs and easy access. They have high similarities with humans in terms of tissue structure, metabolism, and immune system, and are therefore often used to study tissue healing. This article selected 60 mice with similar human sports injuries, weighing 20–25 g; the mice were randomly divided into two groups, regardless of gender. Composite conjugated materials were implanted into specific tissues or organs. The control group was implanted with non conjugated materials, while the experimental group was implanted with conjugated materials. The experimental period was 5 weeks, and the effects of different materials on the tissue healing process were observed and compared.

### 4.2 Material characteristic experiment

(1) Comparison of biocompatibility

Biocompatibility includes tissue compatibility and blood compatibility. Biocompatibility refers to the ability of a material to come into contact with living tissue and body fluids without causing a decrease in cell or tissue function, and to prevent inflammation, cancer, or rejection reactions in the tissue. This article observed the biocompatibility of the control group and experimental group in terms of adverse reactions, decreased cell function, decreased tissue function, and inflammation after implantation of materials. The observation results of the control group are shown in Table 1.

**TABLE 1:** Observation results of the control group (multiple choices).

Index	Number of mice	Percentage(%)
Rejection reaction	15	50
Decreased cellular function	12	40
Decreased tissue function	9	30
Inflammation occurs	15	50

In Table 1, 15 mice in the control group experienced rejection reactions, with a percentage of 50%; 12 mice showed a decrease in cellular function, with a percentage of 40%; 9 mice showed a decrease in tissue function, with a percentage of 30%; 15 mice developed inflammation, with a percentage of 50%.

The observation results of the experimental group are shown in Table 2.

**TABLE 2:** Observations of the experimental group (multiple choices).

Index	Number of mice	Percentage(%)
Rejection reaction	5	16.7
Decreased cellular function	3	10
Decreased tissue function	1	3.3
Inflammation occurs	5	16.7

In Table 2, 5 mice in the experimental group experienced rejection reactions, with a percentage of 16.7%; 3 mice showed a decrease in cellular function, with a percentage of 10%; 1 mouse showed a decrease in tissue function, with a percentage of 3.3%; 5 mice developed inflammation, with a percentage of 16.7%.

Composite conjugated materials are better biocompatible than ordinary materials, which is due to their biodegradability, physicochemical properties, cytocompatibility, immunocompatibility and hemocompatibility.

(2) Comparison of antibacterial effects

Composite conjugated materials also have antibacterial properties, which can kill bacteria and other microorganisms and prevent infection. When a wound occurs, bacteria and other microorganisms around the wound usually invade the wound, easily causing infection and delayed healing. By applying composite conjugated materials with antibacterial properties to the wound site, microorganisms can be eliminated and infection can be prevented, thereby promoting wound healing.

**FIGURE 4:**
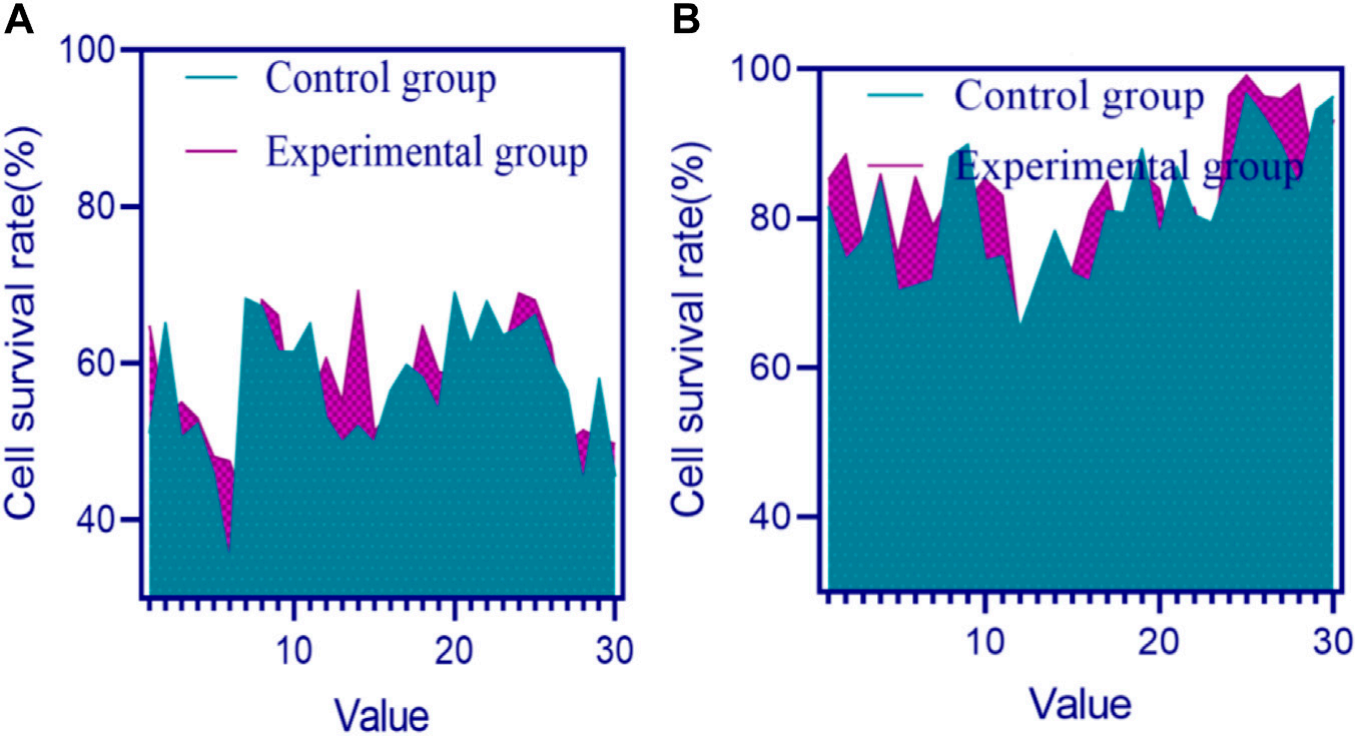
Cell survival rates of the control group and experimental group before and after implantation of materials **(A)** Cell survival rates of two groups before implantation of materials **(B)** Cell survival rates of two groups after implantation of materials.

A higher cell survival rate means that the material has lower cytotoxicity to cells, which means that the antibacterial effect is better. The cell survival rates of the control group and experimental group before and after implantation of the material are shown in Figure 4 (in Figure 4, the horizontal axis represents the number of mice, which is a numerical value, and the vertical axis represents the cell survival rate).

In Figure 4A, the overall cell survival rate in the control group and experimental group mice before material implantation was below 80%. In Figure 4B, the highest cell survival rate in the control group and experimental group mice exceeded 90% after implantation of the material.

The overall cell survival rate in the experimental group mice was higher than that in the control group. By applying composite conjugated materials with antioxidant properties to the wound site, cells can be protected from oxidative damage and wound healing can be accelerated.

### 4.3 Effect of tissue healing

(1) Skin healing analysis

Evaluating the degree of skin healing through drug release rate can achieve sustained and appropriate provision of drugs, which can promote the repair and healing process of wounds, and effectively control the occurrence of infections, inflammatory reactions, and side effects, thus evaluating and intervening in skin healing.

**FIGURE 5:**
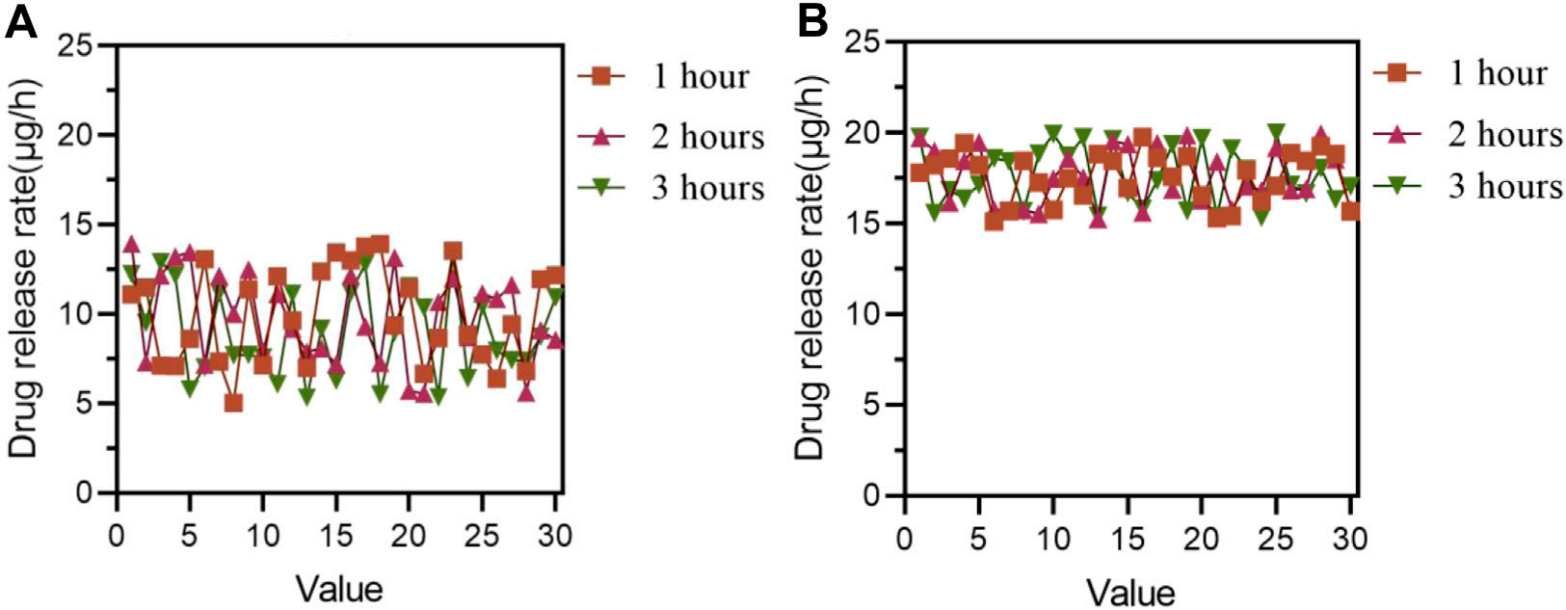
Drug release rates in the control and experimental groups **(A)** Drug release rate in the control group **(B)** Drug release rate of the experimental group.

The materials implanted into two groups of mice included relevant drugs, with drug release rates generally ranging from 5 to 20 μg per hour, μg/h); the higher the range, the better. The drug release rates of the control and experimental groups are shown in Figure 5 (in Figure 5, the horizontal axis represents the number of mice, which is a numerical value, and the vertical axis represents the drug release rate).

In Figure 5A, the overall range of drug release rate in the control group of 30 mice after medication was within 15 μg/h; in Figure 5B, the drug release rate of the experimental group of 30 mice after medication mostly ranged from 15 to 20 μg/h.

In Figure 5, it was found that the drug release rate of the experimental group of 30 mice after medication was generally higher than that of the control group of 30 mice after medication.

In sports rehabilitation training, due to external stimuli such as greater friction, pressure, and stress that the skin may experience during the exercise process, it is difficult to completely rely on traditional drugs to accelerate the healing of large or deep skin injuries. At this point, more functional materials and other auxiliary means may be needed to assist the skin healing process.

The skin healing degree of the two groups after the experiment is shown in Table 3.

**TABLE 3:** Skin healing degree of two groups after the experiment (%).

Week	Control group	Experimental group
1	42.55	58.17
2	46.11	63.20
3	47.26	66.42
4	49.02	69.96
5	51.28	73.24

In Table 3, the skin healing rates of the control group and the experimental group were 42.55% and 58.17%, respectively, 1 week after the experiment; after 5 weeks of the experiment, the skin healing rates of the control group and the experimental group were 51.28% and 73.24%, respectively.

The skin healing degree of both the experimental group and the control group increased with time, but the skin healing degree of the experimental group was always higher, indicating that the materials in the experimental group are more conducive to skin healing.

(2) Fracture healing analysis

**FIGURE 6:**
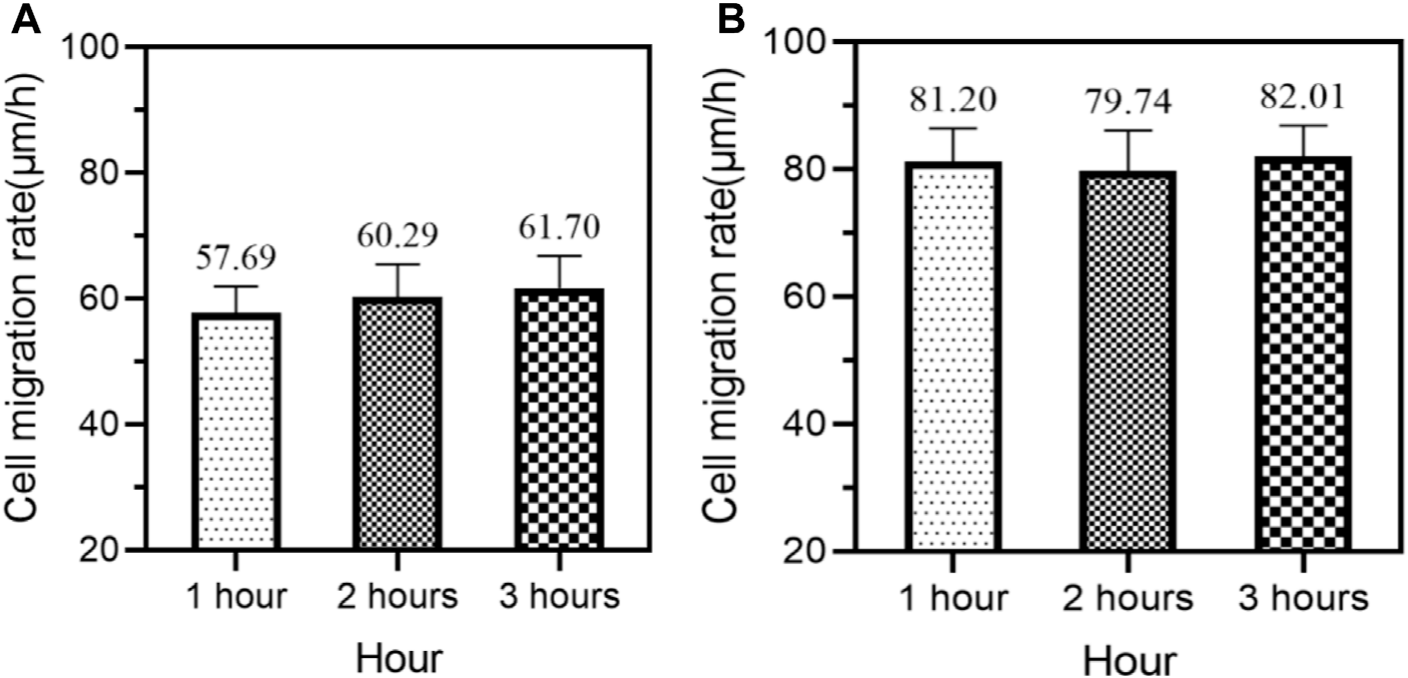
Average cell migration rates of the control group and experimental group at different times after medication **(A)** Mean cell migration rate of the control group **(B)** Average cell migration rate of the experimental group.

The higher cell migration rate means that composite conjugated materials can effectively promote cell movement and wound closure. The unit of mobility is usually micrometer per hour (μm/h), which generally ranges from 60 to 100 μm/h. Due to the large amount of time spent in the previous experiment, only 10 mice in each group were selected for analysis of cell migration rate. The average cell migration rate of the control group and experimental group at different times after medication is shown in Figure 6 (the horizontal axis of Figure 6 represents time, and the vertical axis represents cell migration rate).

In Figure 6A, the average cell migration rates of the control group of 10 mice at 1, 2, and 3 h of medication were 57.69 μm/h, 60.29 μm/h, and 61.70 μm/h, respectively.

In Figure 6B, the average cell migration rates of the experimental group of 10 mice at 1, 2, and 3 h of medication were 81.20 μm/h, 79.74 μm/h, and 82.01 μm/h, respectively.

In Figure 6, regardless of whether after 1 or 2 h of medication, the average cell migration rate of the experimental group of 10 mice was higher than that of the control group of 10 mice.

The degree of fracture healing after the experiment is shown in Table 4.

**TABLE 4:** Fracture healing degree after the experiment.

Week	Control group	Experimental group
1	52.81	65.74
2	51.66	71.64
3	55.23	79.71
4	62.56	83.31
5	67.34	87.94

In Table 4, 1 week after the experiment, the fracture healing rates of the control group and the experimental group were 52.81% and 65.74%, respectively; after 5 weeks of the experiment, the fracture healing rates of the control group and the experimental group were 67.34% and 87.94%, respectively.

As time went on, the fracture healing degree of both the control group and the experimental group gradually increased, but the fracture healing degree of the experimental group was significantly higher than that of the control group.

(3) Analysis of soft tissue damage repair

**FIGURE 7:**
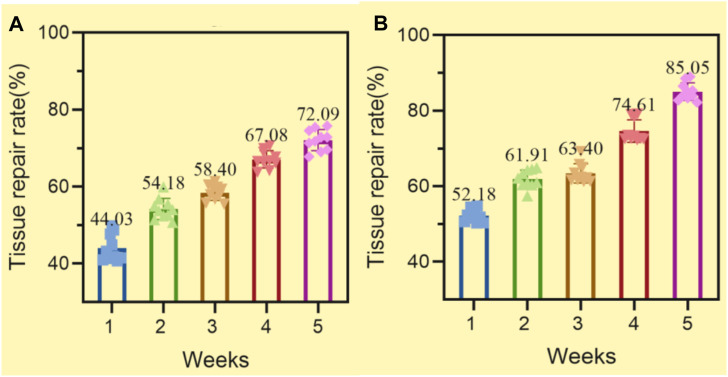
Average tissue repair rate of the control and experimental groups **(A)** Tissue repair rate in the control group **(B)** The tissue repair rate of the experimental group.

The higher the tissue repair rate, the better. As time increases, the tissue repair rate would increase, reaching a maximum of 100%. The average tissue repair rates of the control group and experimental group after 5 weeks of experiment are shown in Figure 7 (where the horizontal axis represents week and the vertical axis represents tissue repair rate).

In Figure 7A, after 1, 2, 3, 4, and 5 weeks of the experiment, it was found that the average tissue repair rates of the control group were 44.03%, 54.18%, 58.40%, 67.08%, and 72.09%, respectively. As time went on, the average tissue repair rate of the control group also increased, indicating that soft tissue damage in mice was gradually improving.

In Figure 7B, after 1, 2, 3, 4, and 5 weeks of the experiment, it was found that the average tissue repair rates in the experimental group were 52.18%, 61.91%, 63.40%, 74.61%, and 85.05%, respectively.

From Figure 7, it can be seen that after 1, 2, 3, 4, and 5 weeks of the experiment, the average tissue repair rate of the experimental group was higher than that of the control group.

## 5 Discussion

From Tables 1, 2, it can be seen that the materials implanted in the experimental group of mice have better biocompatibility. The structure of composite conjugated materials can regulate inflammatory reactions by adjusting the porosity and surface morphology of the material, and control the diffusion and adsorption of cells and molecules by adjusting the porosity and pore size of the material, thereby affecting cell adhesion, migration, and proliferation. In addition, the surface morphology and chemical properties of materials can be adjusted to alter the interaction between materials and cells, thereby regulating cell activity and inflammatory response.

Composite conjugated materials are usually biodegradable and can gradually degrade within tissues and be metabolized out of the body. This biodegradability prevents the material from accumulating in the body, reducing potential toxic side effects on the body. The special structure and composition of materials can simulate the characteristics of natural tissues and stimulate cell proliferation and differentiation, thereby promoting tissue regeneration and repair. Composite conjugated materials typically have good compatibility when in contact with blood. They can reduce platelet adhesion and activation, reducing the risk of thrombosis. In addition, the special structure and surface properties of the material can also reduce the interaction between the material and blood components, and improve blood compatibility. The good biocompatibility of composite conjugated materials has greater potential for their application in the biomedical field, which can better meet the needs of tissue regeneration and repair.

Figure 5 shows that the drug release rate of the experimental group was higher than that of the control group. Composite conjugated materials are typically composed of multiple components, some of which may have higher porosity or surface area, thus providing more drug storage space. In this way, the contact area between the drug and the material increases, which promotes the faster release of the drug. The structural design of composite conjugated materials can also affect the drug release rate. For example, by adjusting the pore structure, surface morphology, and other parameters of the material, the diffusion rate of drugs in the material can be changed, thereby affecting the drug release rate.

Table 3 shows that composite conjugated materials are more effective in promoting skin healing. Composite conjugated materials have good wetting properties, helping to form a suitable wetting environment for wounds and promoting the healing process. In addition, composite conjugated materials with active ingredients such as controlled release drugs or growth factors can release the substances required for treatment on the wound surface, accelerating the healing process. For larger or deeper wounds, it may be necessary to use composite conjugated materials in sports rehabilitation training to accelerate skin healing. For specific treatment plans and material selection, it is recommended to consult a professional doctor or rehabilitation specialist and make decisions based on the specific situation. Composite conjugated materials promote cell movement, material exchange, and healing processes on the wound surface. By adding active ingredients such as drugs or growth factors to the composite material and regulating the release rate, drugs can be continuously or gradually released. This can provide the necessary bioactive substances for the wound surface within a certain period of time, thereby promoting cell proliferation, neovascularization, and collagen deposition during the healing process.

Table 4 found that the experimental group had a higher degree of fracture healing. Composite conjugated materials can promote fracture healing through internal and external applications. Through internal applications, composite conjugated materials can serve as scaffolds or fillers to promote bone tissue regeneration. Composite conjugated materials can provide the necessary support and growth environment for bone tissue, thereby promoting bone tissue regeneration and repair. Through external applications, composite conjugated materials can be used as patches or dressings to promote wound surface healing. Composite conjugated materials can attach to the surface of the skin and slowly release drugs, thereby helping to promote bone tissue regeneration and repair.

Figure 7 shows that as time increased, the average tissue repair rate in the experimental group increased by a greater extent than that in the control group, indicating that the materials in the experimental group are more conducive to the recovery of soft tissue damage. Composite conjugated materials can promote vascular regeneration, which is crucial for wound healing. When a wound occurs, blood vessels are usually damaged and blood flow is obstructed, leading to tissue necrosis and delayed wound healing (Dubois et al., 2020). By applying composite conjugated materials with angiogenesis ability to the wound site, vascular regeneration can be accelerated, thereby promoting wound healing. Composite conjugated materials can improve the safety of exercise rehabilitation training, which is a highly secure rehabilitation method. Composite conjugated materials can improve the safety of exercise rehabilitation training and reduce the risks during the training process. Composite conjugated materials can provide better support and protection, reducing the likelihood of injury.

## 6 Conclusion

With the continuous progress of technology and the continuous development of medical technology, composite conjugated materials in the field of sports rehabilitation training have become a highly concerned technology. The role of conjugated materials in tissue healing during exercise rehabilitation training is very important. It promotes tissue repair and regeneration and can improve the effectiveness of sports rehabilitation training, thus increasing the safety of sports rehabilitation training. This material can play an important role in tissue healing and provide effective support for wound treatment. After the experiment in this article, it was found that composite conjugated materials not only have better biocompatibility and antibacterial effects compared to ordinary materials, but also have higher effects on skin healing and tissue repair than ordinary materials. Composite conjugated materials can not only accelerate tissue healing, but also reduce side effects during the treatment process, thereby improving the effectiveness of wound treatment. With the continuous evolution of composite conjugated materials, their future development prospects would be even broader.

## Data Availability

The original contributions presented in the study are included in the article/Supplementary material, further inquiries can be directed to the corresponding author.
